# Genetic and environmental causes of variation in gestation length of Jersey crossbred cattle

**DOI:** 10.14202/vetworld.2016.351-355

**Published:** 2016-04-06

**Authors:** Anshuman Kumar, Ajoy Mandal, A. K. Gupta, Poonam Ratwan

**Affiliations:** 1 Dairy Cattle Breeding Division, ICAR-National Dairy Research Institute, Karnal, Haryana, India; 2 Animal Breeding Section, Eastern Regional Station, ICAR-National Dairy Research Institute, Kalyani, West Bengal, India

**Keywords:** crossbred cattle, genetic and environmental factors, gestation length, heritability

## Abstract

**Aim:**

The objective of this study was to investigate the effect of genetic and non-genetic factors and estimate the genetic parameter for gestation length (GL) of Jersey crossbred cattle.

**Materials and Methods:**

The data included the 986 parturition records on Jersey crossbred cattle maintained at the Eastern Regional Station of ICAR-National Dairy Research Institute, Kalyani, West Bengal, India during 36 years (1978-2013). The data were analyzed applying mixed model least square technique considering the fixed effects of genetic group, season of calving, period of calving, parity of animal, birth weight, and sex of calf born from animal. The effect of sire was included as a random effect in the model.

**Results:**

The genetic group of animal, season of calving, parity of animal, and birth weight of calf born were found to be a significant source of variation in the GL, whereas the period of calving and sex of calf did not affect this trait. Cows with <50% and >62.5% Jersey inheritance had the shortest and longest GLs, respectively. Cows calved in summer and rainy season had shorter GL than those calved in the winter season. Older cows in 4^th^ parity carried calves for longer days than the cows in 1^st^ parity. The increase in calf birth weight significantly (p<0.01) contributed to a linear increase in GL value in this study. The heritability estimate of GL was 0.24±0.08.

**Conclusion:**

It can be concluded that selection for lower GL without distressing future growth of calf can be used to reduce calving difficulty, but a very small standard deviation of GL limits the benefit. Moreover, more accurate prediction of calving date will help in better management and health care of pregnant animals.

## Introduction

Gestation length (GL) is one the most important traits in cow-calf operations and significantly affects cattle breeding and production. Although the phenotypic variation of GL is biologically limited and has no direct economic benefit as such, many reports have shown its high genetic correlations with birth weight [1] and dystocia [2]. Calving difficulty in cows undermines the economic viability of dairy herds due to extensive calf losses, production of weak calves, and huge veterinary cost. Furthermore, additional losses result from impaired reproductive performance of cow calved with difficulty which takes longer time and increased number of inseminations to conceive.

GL has some prospects for selection to reduce calving problems as it has moderate to high heritability [3]. Moreover, selection for shorter gestation periods will decrease the rearing period for heifers and the calving interval for cows. GL has so far received little or no attention in dairy farm operations, and there is scanty information available on genetic evaluation of this trait under Indian conditions. Furthermore, a comprehensive study regarding variation in GL with environment and level of exotic inheritance in crossbred cattle of India is lacking.

Therefore, the objective of this study was to determine the various genetic and non-genetic factors influencing GL and the estimation of the genetic parameter in Jersey crossbred dairy cattle maintained at the dairy farm of this institute. Since animals with different levels of Jersey inheritance exist; the present study was also carried out to analyze the variation in GL with genetic grades. Establishing the range of these variations is economically important, as it may reduce the costs of parentage control of individuals born out of particularly long pregnancies [4].

## Materials and Methods

### Ethical approval

The present study was approved by the Institutional Animal Ethics Committee of National Dairy Research Institute (NDRI).

### Location of study and management of animal

The study was carried out at Eastern Regional Station of ICAR-NDRI, Kalyani, Nadia, West Bengal, India. Kalyani is located in the lower Gangetic basin of West Bengal. The farm is located at an average altitude of 9.75 m above the mean sea level on 22.59’N latitude and 88.29’E longitude. The climatic conditions and management practices at this station have been previously described [5]. Animals were raised under zero grazing loose housing system, and the nutritional requirements of the cows were met through a standardized ration of concentrate and *ad libitum* green fodder.

### Sample population, classification, and editing of data

Insemination and calving records of Jersey crossbred cattle kept at Eastern Regional Station of ICAR-NDRI, Kalyani, Nadia, West Bengal, India, calved during a period of 36 years (1978-2013) were used. The information was collected from AI records and reproduction sheets maintained at different sections of institute, *viz*., record room of Animal breeding section and Cattle Yard, ERS of ICAR-NDRI, Kalyani. GL was determined as the interval from the date of the last insemination to the date of subsequent calving. To ensure the normal distribution, the outliers were removed, and data within the range of mean±2 standard deviation were only considered. Since there were animals with different levels of Jersey inheritance, the genetic group of each animal was deduced after back tracing of the pedigree of animal. To evaluate the effect of various genetic and non-genetic factors on GL, the data were grouped into different classes to be used as fixed effects. The data were classified as shown in Table-1. The GL of heifers was assigned first parity in this study. The random effect of sire was also included in the model. Only those gestations which terminated in single births of normal calves were considered in the study. Moreover, animals with abortion, stillbirth, or premature birth records were not included in the present study. For the genetic studies, sires having three or more daughters were only considered.

**Table-1:** Classification of data for genetic and environmental factors.

Genetic group (8 classes)
<50% J
50% J-50% T
50% J-50% RS
50% J-25% T-25% RS
Misc. 50% J
51%-62% J
62.5% J
>62.5% J
Season of calving (3 classes)
Winter (November-February)
Summer (March-June)
Rainy (July-October)
Period of calving (6 classes)
Before 1989
1989-1993
1994-1998
1999-2003
2004-2008
2009-2013
Parity
1
2
3
4
5
6 and above
Birth weight of calf born (6 classes) (kg)
≤16
1-6.119
1-9.122
2-2.125
2-5.128
>28
Sex of calf born (2 classes)
Male
Female

J=Jersey, T=Tharparkar, RS=Red Sindhi, Misc.=Miscellaneous

### Statistical analysis

The effects of genetic and non-genetic factors on reproductive traits were carried out by least square analysis of variance using the technique described by Harvey [6]. Duncan’s multiple range test as modified by Kramer [7] was used for testing the differences between least squares means between sub-classes. Genetic parameters were estimated using Model 2 of Mixed Model Least square and Maximum Likelihood, PC-2 Version Computer Program [6].

The following model was used:

Y_ijklmnop_= µ+S_i_+(GG)_j_+(Sea)_k_+P_l_+(Pa)_m_+(Bw)_n_ (Sex)_o_+e_ijklmnop_

Where,

Y_ijklmnop_= Phenotypic value of trait on the p^th^ animal

m=Overall mean

S_i_=Random effect of i^th^ Sire

(GG)_j_=Fixed effect of j^th^ Genetic group (1-8)

(Sea)_k_=Fixed effect of k^th^ season of calving (1-3)

P_l_=Fixed effect of l^th^ period of calving (1-6)

(Pa)_m_=Fixed effect of m^th^ parity of cow (1-6)

(Bw)_n_=Fixed effect of birth weight of calf born (1-6)

(Sex)_o_=Fixed effect of sex of calf born (1-2)

e_ijklmnop_=Random error ɾ NID (0, σ^2^_e_)

## Results and Discussion

**Figure 1:**
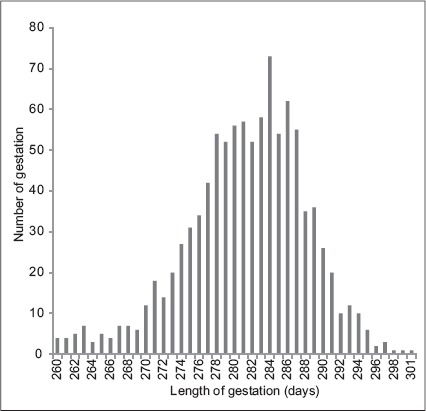
Frequency distribution of gestation length in Jersey crossbred cattle.

Frequency distribution of the GL is presented in Figure-1 - The curve is skewed toward a length of gestation of over 285 days. The mean GL recorded in Jersey crossbred cattle was 280±0.25 days (Table-2), with a range of 260-301 days and coefficient of variation of 2.19%. It is in agreement with the other reports in Jersey crossbred cattle [8,9]. Norman *et al*. [10] also reported similar results for GL with mean value of 280 days for Jersey cattle. However, the mean GL of animals in this study was higher than the finding of Bhutkar *et al*. [11], Mondal *et al*. [12], and Varaprasad *et al*. [13] who obtained the values of 274.93, 275, and 276.89 days, respectively, in crossbred cattle of India.

**Table-2:** Degrees of freedom, mean squares, F values and significance of sources of variation affecting gestation length in Jersey crossbred cattle.

Source of variation	Degrees of freedom	Mean square	F value	p
Sire	39	81.60	2.30	0.0000
Genetic group	7	119.63	3.37	0.0016
Season	2	254.45	7.16	0.0008
Period	5	32.10	0.90	0.4796
Parity	6	97.99	2.76	0.0176
Birth weight of calf	5	1131.38	31.83	0.0000
Sex of calf	1	67.23	1.89	0.1694
Error	921	35.54		

### Effect of sire

Sire had highly significant (p<0.01) effect on length of gestation of crossbred cattle in the present study (Table-2).

### Effect of genetic group

The gestation period of animals was significantly (p<0.01) varied among the animals of different genetic groups in this study (Tables-2 and 3) which was in conformity with the findings of Norman *et al*. [10], who reported that GL is typical for breed in their study on different breeds of cattle. The present study revealed that cows with <50% and >62.5% Jersey inheritance had the shortest (279.10±1.01 days) and longest (283.12±0.94 days) gestation periods, respectively. The results were almost similar to Bahmani *et al*. [14], who reported lowest GL to 25% exotic inheritance group, whereas the highest value was related to 50% and ≥75% group. The longer GL of animals having Jersey inheritance of more than 62.5% in the current study may be due to the more milk production potential of cows with more exotic inheritance as there may be an existence of a positive correlation between milk yield and GL of animals [15]. However, the present findings were different from Mondal *et al*. [12], who reported the non-significant influence of genetic group on GL of animals.

**Table-3:** Least squares means (±SE) for GL of Jersey crossbred cattle.

Parameters	N	GL (days)
Overall mean	986	280.25±0.54
Genetic group		
<50% J	78	279.10^c^±1.01
50% J-50% T	182	279.76^c^±1.09
50% J-50% RS	82	281.36^ab^±0.86
50% J-25% T-25% RS	133	279.35^c^±0.84
Misc. 50% J	280	279.47^c^±0.65
51%-62% J	80	279.23^c^±0.93
62.5% J	63	280.64^bc^±1.00
>62.5% J	78	283.12^a^±0.94
Season		
Winter (November-February)	305	281.16^a^±0.62
Summer (March-June)	333	280.29^ab^±0.61
Rainy (July-October)	348	279.31^b^±0.60
Period		
Before 1989	69	278.81±1.60
1989-1993	109	278.84±1.21
1994-1998	169	279.62±0.83
1999-2003	181	280.14±0.83
2004-2008	219	282.02±1.07
2009-2013	239	282.09±1.42
Parity		
1	253	279.65^b^±0.69
2	232	280.30^ab^±0.66
3	171	281.28^a^±0.69
4	119	281.31^a^±0.76
5	87	279.45^b^±0.85
6 and above	124	279.54^b^±0.88
Birth weight of calf (kg)		
≤16	45	274.49^e^±1.05
16.1-19	100	276.62^d^±0.80
19.1-22	251	280.00^c^±0.62
22.1-25	322	282.01^b^±0.60
25.1-28	198	284.03^a^±0.66
>28	70	284.38^ab^±0.89
Sex of calf		
Male	512	280.53±0.58
Female	474	279.98±0.58

Means with different superscripts in columns within fixed effects differ significantly (p<0.05). GL=Gestation length, SE=Standard error, J=Jersey, T=Tharparkar, RS=Red Sindhi

### Effect of season of calving

The influence of season of calving was significant (p<0.01) with cows calved in summer and rainy season had shorter GL than those calved in the winter season (Tables-2 and 3). Similar findings were reported by Petrović *et al*. [16], Bakir *et al*. [17], and Melaku *et al*. [18], who reported that shorter GL were associated with high summer temperature. In contrast, Silva *et al*. [15] found no difference in GL between warm and cool seasons in Florida. The shorter GL in summer and rainy season in the present study may be attributable to dietary changes and high temperature during the last phase of gestation which speeds up the parturition process [19].

### Effect of period of calving

The period of calving of animal had no significant influence on GL in this study (Table-2). The present results were in agreement with Turkyilmaz [20], who reported the non-significant difference between GLs of Holstein cows calved in different years. However, there was a total linear change of 3.28 days over 39 years. This finding is quite similar to Silva *et al*. [15] who noticed a significant increase of 4 days over a period of 50 years in GL of Guernsey, Holstein, and Jersey cows at several Florida-based farms. Bhutkar *et al*. [11] also reported a significant effect of period on GL in crossbred cattle. This gradual increase in GL over the years could be attributed to the sharp rise in production level.

### Effect of parity

In the present study, GL of animals was significantly (p<0.05) influenced by parity of animal at breeding (Table-2). Animals in 4^th^ parity had the longest GL (281.31±0.76 days), whereas heifers and cows of 5^th^ parity onward had the lowest GL. This study was similar to the study of Petrović *et al*. [16] and Nogalski and Piwczyński [21] who reported a longer gestation period in older cows with a significant difference of 1 day between 1^st^ and 3^rd^ calving. Results of the present study were also consistent with earlier studies [17,19,22] on crossbred cattle, who observed that parity of dam significantly affects GL in cattle with heifers having shorter GL than cows. Moreover, older cows carried their calves for longer days than younger cows because of the relatively large uterus. However, Messine *et al*. [23] and Menon *et al*. [24] reported no difference in GL between heifers and cows.

### Effect of birth weight of calf

The increase in calf birth weight significantly (p<0.01) contributed to a linear increase in GL value in this study (Table-3). In the dams of heaviest calves (>28 kg), on an average GL of animals was 9.54 days longer than dams with lightest calves (≤16 kg). The results were consistent with findings of Nogalski and Piwczyński [21] and Nadarajah *et al*. [25] who reported a difference of 3.7 and 3.9 days, respectively, between cows with large and small fetuses. Cows with heavier calves could result in increased risk of dystocia and stillbirth [26,27].

### Effect of sex of calf

The present study revealed the non-significant effect of sex of calf on GL of the dam. Similar non-significant effects of sex of calf on GL were noticed by Melaku *et al*. [18]. However, several published reports were in disagreement with the present study, which showed that sex of calf significantly affects GL, with male calves gestated 1-3 days longer than females [10,24,26].

### Heritability estimate

Heritability for GL of Jersey crossbred cattle in the current study was 0.24±0.08 within the range of previous studies [15,21] who estimated the heritability of 0.22 and 0.21, respectively, but the estimate lower than this value was reported by Johanson *et al*. [28]. The moderate heritability suggests the genetic possibility of changing the trait. However, selection for GL is undesirable, as extreme values of this trait may lead to calving difficulties and stillbirth [29]. Furthermore, the possibility of reducing the inter-calving period through a selection of shorter GL is little due to small genetic standard deviation.

## Conclusions

The genetic group of animal, season of calving, parity of animal, and birth weight of calf were found to be significant sources of variation in the GL of the Jersey crossbred cattle, whereas the period of calving and sex of calf did not affect this trait. Cows with <50% and >62.5% Jersey inheritance had the shortest and longest GLs, respectively. Shorter GL were also associated with the high temperature of summer and rainy season. Older cows carried calves for a longer period than did younger cows; however, it was not true for extremely old cows. Calves born from shorter GL present lower birth weight, which could result in less calving difficulty. Thus, the selection for lower GL without distressing future growth traits in dairy cattle improvement program can be used to reduce dystocia which will further increase the probability of number of live calves born in the herd. However, in spite of higher heritability of GL, the standard deviation of GL was very small which limit the benefit of selection for GL. Moreover, selection for GL should be done with caution as both longer and shorter gestation periods contribute to a higher number of stillbirths [21]. More accurate prediction of calving dates can help dairy producers to meet management requirements of pregnant animals and to administer better healthcare during high-risk phases of animals’ lives.

## Authors’ Contributions

AM has planned the study. AK recorded the information and analyzed the data. AKG acted as an advisory committee member and contributed immensely right from the start to end of the experiment. PR provided help in the analysis of data. AK drafted and revised the manuscript under the guidance of AM. All authors read and approved the final manuscript.
